# Identification of host proteins interacting with *Toxoplasma gondii* GRA15 (TgGRA15) by yeast two-hybrid system

**DOI:** 10.1186/s13071-016-1943-1

**Published:** 2017-01-03

**Authors:** Qing Liu, Fa-Cai Li, Hany M. Elsheikha, Miao-Miao Sun, Xing-Quan Zhu

**Affiliations:** 1 College of Veterinary Medicine, Hunan Agricultural University, Changsha, Hunan Province 410128 People’s Republic of China; 2 State Key Laboratory of Veterinary Etiological Biology, Key Laboratory of Veterinary Parasitology of Gansu Province, Lanzhou Veterinary Research Institute, Chinese Academy of Agricultural Sciences, Lanzhou, Gansu Province 730046 People’s Republic of China; 3 Faculty of Medicine and Health Sciences, School of Veterinary Medicine and Science, University of Nottingham, Sutton Bonington Campus, Loughborough, LE12 5RD UK; 4 Jiangsu Co-innovation Center for Prevention and Control of Important Animal Infectious Diseases and Zoonoses, Yangzhou, Jiangsu Province 225009 People’s Republic of China

**Keywords:** *Toxoplasma gondii*, TgGRA15, Host-pathogen interaction, Yeast-two-hybrid, Luzp1, AW209491

## Abstract

**Background:**

*Toxoplasma gondii*, an obligate intracellular protozoan parasite, possesses the remarkable ability to co-opt host cell machinery in order to maintain its intracellular survival. This parasite can modulate signaling pathways of its host through the secretion of polymorphic effector proteins localized in the rhoptry and dense granule organelles. One of such effectors is *T. gondii* type II-specific dense granule protein 15, TgGRA15, which activates NF-κB pathway. The aim of the present study was to identify the host interaction partner proteins of TgGRA15.

**Methods:**

We screened a yeast two-hybrid mouse cDNA library using TgGRA15 as the bait. TgGRA15 (PRU strain, Type II) was cloned into the pGBKT7 vector and expressed in the Y2HGold yeast strain. Then, the bait protein expression was validated by western blotting analysis, followed by auto-activation and toxicity tests in comparison with control (Y2HGold yeast strain transformed with empty pGBKT7 vector).

**Results:**

This screening led to the identification of mouse Luzp1 and AW209491 as host binding proteins that interact with TgGRA15. Luzp1 contains three nuclear localizing signals and is involved in regulating a subset of host non-coding RNA genes.

**Conclusions:**

These findings reveal, for the first time, new host cell proteins interacting with TgGRA15. The identification of these cellular targets and the understanding of their contribution to the host-pathogen interaction may serve as the foundation for novel therapeutic and prevention strategies against *T. gondii* infection.

## Background

*Toxoplasma gondii* infection is a global public health challenge with approximately one third of the human population reported as chronic carriers [1]. This parasite infects and reproduces in virtually any nucleated cell of warm-blooded animals [2–4]. The disease that *T. gondii* causes in humans ranges from asymptomatic to debilitating or even fatal consequences in AIDS patients, organ transplants and during pregnancy [5, 6]. In the last two decades, significant progress has been made towards a better understanding of the factors involved in the pathogenesis of *T. gondii*. These advances include the identification of several parasite-derived effector proteins, such as ROPs and GRAs from the parasite rhoptry and dense granule organelles, respectively. These effectors are essential for *T. gondii* invasion and colonization [7–9]. For instance, GRAs direct the translocation into host cells of a battery of effector proteins, which modulate a variety of cellular processes including the maintenance of the parasitophorous vacuole (PV), gene expression, vesicle trafficking, and programmed cell death [7–14]. Modulation of these cellular processes allows *T. gondii* to replicate within host cells, avoid host defense mechanisms and ultimately sustain its intracellular survival.

About 30 of GRAs, such as TgGRA3, TgGRA5, TgGRA6, TgGRA7, TgGRA15, TgGRA16 and TgGRA18 have been identified [11, 15–18]. However, the exact roles of these proteins in the intracellular survival and growth of *T. gondii* are not completely understood. Of interest is the dense-granule protein GRA15 of Type II *T. gondii* strains, TgGRA15, which activates NF-κB signaling pathways most likely through activation of TRAF6, which in turn activates IκB kinase (IKK), leading to the phosphorylation and degradation of IκB, facilitating the subsequent translocation of NF-κB to the nucleus to induce a broad range of genes involved in the immune response and inflammation [19, 20]. Also, TgGRA15 induces classical activation of macrophages [10, 11, 13, 21]. These functions are critical in innate immune defense mechanism [13, 22–24]. TgGRA15 can also affect the accumulation of p65 guanylate binding protein 1 (GBP1) on the PV in the affected murine cells [25]. Even though TgGRA15 plays key roles in *T. gondii* pathogenesis, information of the interaction of TgGRA15 with TRAF6, IKK or murine GBP1 (mGBP1) protein remains hypothetical and the specific host cell interaction partner proteins of TgGRA15 are still unknown.

The advent of yeast-based screening assays, such as the yeast two-hybrid system [26, 27] has resulted in a considerable increase in the number of protein interactions reported in the scientific literature because these assays allow rapid discovery of new protein interactions *via* DNA library screening. In the yeast 2-hybrid (Y2H) assay, a given protein is assayed against a mixture of full-length proteins, protein domains and/or protein fragments expressed from a cDNA library, followed by isolation of the protein's interacting partners. What makes Y2H assay a powerful tool for protein-interaction discovery is the lack of the need for prior knowledge of the interaction partners.

To advance the current understanding of the functions of TgGRA15, we attempted to identify host cellular proteins that interact with this parasite protein by means of the yeast two-hybrid system using mouse cDNA libraries. Results indicate that TgGRA15 interacts with Luzp1 and AW209491 host proteins. Identification of these two host proteins interacting with TgGRA15 may significantly improve the understanding of the role of TgGRA15 during *T. gondii* infection.

## Methods

### Parasite strain

*Toxoplasma gondii* (Type II) PRU strain was selected for generating a cDNA library because it encodes the TgGRA15, which allows type II strains of *T. gondii* to be a highly inducer of NF-kB activation, compared to type I strains [28] and type III strains [11]. The Type II PRU strain was maintained in our laboratory by the passage of cysts in female BALB/c mice (4 to 5 weeks of age). Mice were purchased from the Center of Experimental Animals, Lanzhou University, Lanzhou, China. Parasite maintenance in the mice experiment was performed in strict compliance with state and institutional animal care guidelines. The tissue cysts of *T. gondii* PRU strain were harvested from infected mice 30 days post-infection and were used for total RNA extraction.

### Bait plasmid construction

**Fig. 1:**
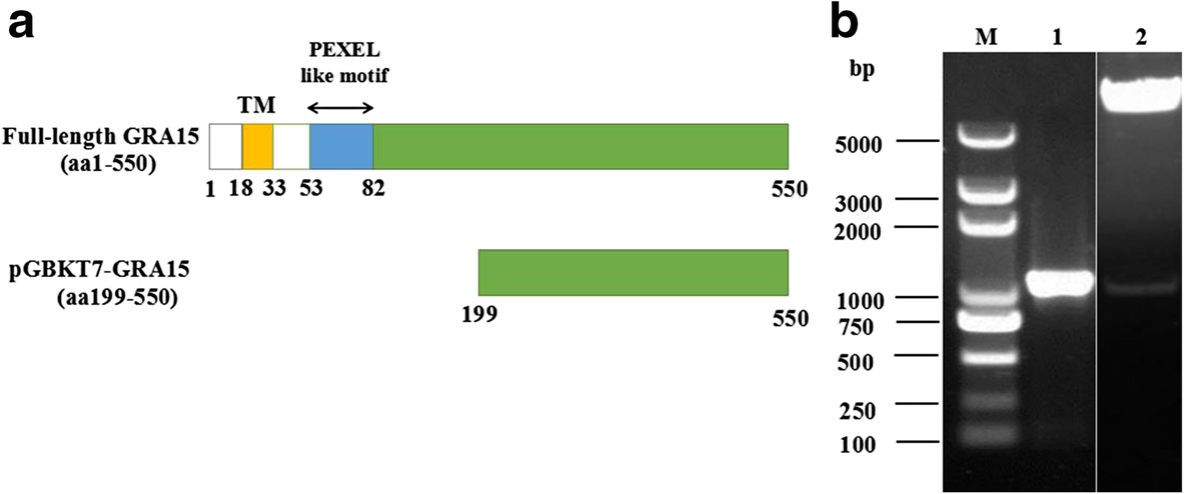
Construction of the pGBKT7-TgGRA15 bait plasmid. **a** Schematic illustration of full-length TgGRA15 and the region of TgGRA15 used in the yeast-two-hybrid screen. **b** Lane M: DL5000 DNA Marker; Lane 1: the gel electrophoresis of the fragment of TgGRA15 amplified from *T. gondii* cDNA; Lane 2: the constructed pGBKT7-TgGRA15 bait plasmid was identified by digestion with EcoRI and BamHI

Total RNA was extracted from parasite cysts by using TRIzol Reagent following the manufacturer’s protocol (Invitrogen, Carlsbad, USA) and was reverse-transcribed into cDNA by using 1st strand cDNA Synthesis kit (TaKaRa, Dalian, China). To construct the TgGRA15 bait plasmid, the TgGRA15 gene fragment encoding a 352-residue (from aa199 to aa550) peptide (Fig. 1a) was PCR amplified from the cDNA by using two gene-specific primers: TgGRA15-F (5'-GAATTCCCATCTACACTCATACCTTCACCAG-3') and TgGRA15-R (5'-GGATCCTCATGGAGTTACCGCTGATTGTGTGTC-3'). The PCR product was ligated into pMD19-T vector (Takara, Dalian, China), which was then digested with restriction enzymes EcoRI and BamHI. The fragment was purified using a StarPrep Gel Extraction Kit (GenStar, Beijing, China), and then ligated into pGBKT7 vector at *Eco*R1 and *Bam*H1 sites. Subsequently, the plasmid (designated pGBKT7-TgGRA15) was transformed into *E. coli* DH5α strain (Transgen, Beijing, China) and verified by endonuclease cleavage and sequencing (Sangon Biotech, Shanghai, China).

### TgGRA15 bait expression in yeast cells

The pGBKT7-TgGRA15 bait plasmid was transformed into yeast strain Y2HGold using the Yeastmaker™ Yeast Transformation System 2 kit (Cat. No. 630439, Clontech, Mountain View, USA). Then transformants were screened on an agar plate containing the minimal yeast medium without tryptophan (SD/-Trp) for 3–5 days. To check the expression of TgGRA15 bait in yeast cells, one colony from the SD/-Trp plate was picked to prepare a 5 ml overnight culture, which was then inoculated into 50 ml YPDA medium and incubated at 30 °C with shaking until the OD600 reached 0.4–0.6. The culture was centrifuged, and total proteins were extracted from the pelleted cells by the Urea/SDS method [29, 30]. Extracted proteins were then separated by 12% SDS-PAGE gels and transferred onto PVDF membrane (Millipore, Billerica, USA). The membrane was incubated with Myc-Tag Mouse mAb (CST, Danvers, USA) for 1 h. Following three 10 min washings in TBST buffer, the membrane was incubated with HRP-conjugated goat anti-mouse secondary antibody (Sigma, St. Louis, USA). The blot was observed using the direct ECL chemiluminescent method (GE, Buckinghamshire, USA). The yeast strain Y2HGold was transformed with pGBKT7-53 using the same method described above and was used as a positive control.

### Autoactivation and toxicity tests

To test autoactivation of the bait protein, the pGBKT7-TgGRA15 and the empty pGBKT7 plasmids were transformed into the yeast strain Y2HGold, respectively. Transformants were then grown on SD/-Trp, SD/-Trp/X-α-Gal (40 μg/ml X-α-Gal) and SD/-Trp/X-α-Gal/AbA (40 μg/ml X-α-Gal and 125 ng/ml Aureobasidin A) agar plates for 3–5 days. The bait that did not autonomously activate the reporter gene was indicated by white colonies on SD/-Trp and SD/-Trp/X-α-Gal plates, and the absence of colonies on SD/-Trp/X-α-Gal/AbA agar plates. Meanwhile, the colony size of Y2HGold transformed with bait plasmid was compared to Y2HGold transformed with the empty pGBKT7 vector. If the bait is toxic, colonies containing the bait vector will be smaller than colonies containing the empty pGBKT7 vector. Only the bait that lacks autoactivation and toxicity was used in the Y2H screening.

### Y2H screening using yeast mating

All yeast strains and reagents for Y2H assays were purchased from Clontech Co. (Mountain View, USA). To identify host cellular proteins that interact with TgGRA15, we screened this protein against a mouse cDNA library. Briefly, the Y187 cells harboring the Mate & Plate™ Universal Mouse (Normalized) cDNA library (Cat. No. 630482, Clontech, Mountain View, USA) cloned into the pGADT7-RecAB vector were used to mate with the Y2HGold cells transformed with pGBKT7-TgGRA15 for 20–24 h at 30 °C. The mated culture was then spread onto SD/-Leu/-Trp/X-α-Gal/AbA plates (DDO/X/A). Blue colonies were patched out onto higher stringency SD/-Ade/-His/-Leu/-Trp/X-α-Gal/AbA (QDO/X/A) plates. To estimate each insert size on potential positive prey plasmids, PCR amplification was performed using Matchmaker™ AD LD-Insert Screening Amplimer Set (Cat. No. 630433, Clontech, Mountain View, USA).

### Rescuing the prey plasmids and confirmation of the interactions

To rescue plasmids from yeast, the prey plasmids in putatively positive hits were isolated using the Easy Yeast Plasmid Isolation Kit (Cat. No. 630467, USA) and were purified by transformation of *E. coli* DH5α competent cells (Transgen, Beijing, China), followed by selection on LB/Amp agar plates and plasmid isolation. To confirm the potential positive interactions, small-scale matings were performed as shown in Table 1. Briefly, each potential positive prey plasmid from the initial screen was transformed into Y187 strain, followed by mating with Y2HGold yeast strain containing pGBKT7-TgGRA15 bait plasmid or empty pGBKT7 vector, respectively. The mated cultures were then spread onto QDO/X/A agar plates. Y2HGold transformed with pGBKT7-53 or pGBKT7 vector were used to mate with Y187 cells containing pGADT7-T, and were used as control.

**Table 1:** Reconfirmation of the potential positive interactions using small-scale mating

Y2HGold yeast strain	Y187 yeast strain							
	Prey1	Prey2	Prey3	Prey4	Prey5	Prey6	Prey7	Prey8
Empty pGBKT7 vector	C1	C2	C3	C4	C5	C6	C7	C8
pGBKT7-GRA15 bait plasmid	1	2	3	4	5	6	7	8

Prey1–8: Prey plasmid isolated from eight blue clones

Prey1–8 were used to transform Y187 cells, respectively, followed by each transformant mating with Y2HGold containing pGBKT7 empty vector to create zygotes (C1-C8)

Prey1–8 were used to transform Y187 cells, respectively, followed by each transformant mating with Y2HGold containing pGBKT7-GRA15 bait plasmid to create zygotes (18)

### Positive prey analysis

The isolated prey plasmids were sequenced by T7 primer and the sequences obtained were blasted against NCBI (National Center for Biotechnology Information) databases to analyze the function of the corresponding mouse genes. Also, based on Gene Ontology (GO) categories, gene functional classification of the identified genes was performed by searching the Mouse Genome Informatics database (http://www.informatics.jax.org/).

## Results

### Construction of the bait plasmid

The fragment of TgGRA15 was successfully amplified from cDNA of *T. gondii* PRU strain, as shown in Fig. 1b, Lane 1. The pGBKT7-TgGRA15 bait plasmid was constructed as described in Methods. The construction of the resultant plasmid was checked and confirmed by restriction enzyme digestion (Fig. 1b, Lane 2).

### Expression, auto-activation and toxicity tests of TgGRA15 bait in yeast cells

**Fig. 2:**
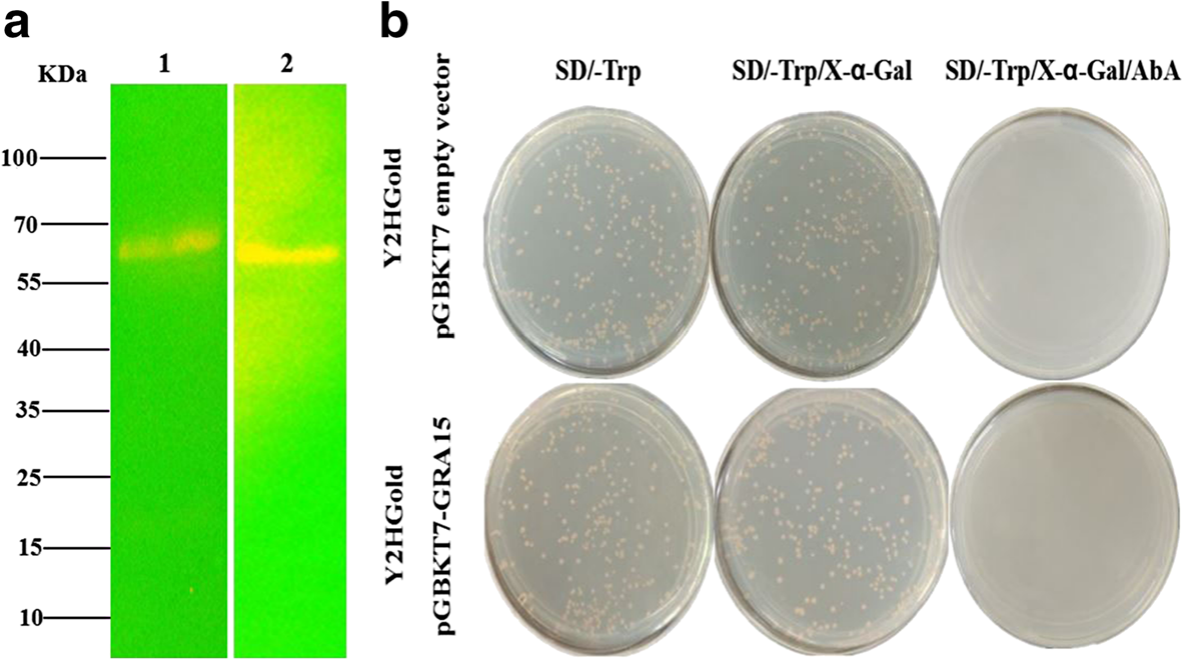
Expression, auto-activation and toxicity tests for the TgGRA15 bait. **a** Western blotting analysis of total protein extracts of Y2HGold containing the following plasmids: Lane 1: pGBKT7-TgGRA15; Lane 2: pGBKT7-53. **b** Determination of the auto-activation and toxicity activity of the pGBKT7-TgGRA15 bait plasmid in yeast cells. The pGBKT7-TgGRA15 bait plasmid and empty pGBKT7 vector were used to transform Y2HGold cells and then grown on different plates

Before Y2H screening, the expression of the fusion protein was confirmed. Total proteins of the yeast strain Y2HGold transformed with pGBKT7-TgGRA15 bait plasmid were extracted and detected by western blotting using the c-Myc epitope tag antibody. The relative molecular weight of the GRA15 bait fusion recognized on the western blot was approximately 57 kDa, which is consistent with its estimated size (Fig. 2a, Lane 1). Y2HGold cells containing the pGBKT7-53 vector that expressed a 57 kDa protein was used as a control (Fig. 2a, Lane 2).

To test the autoactivation activity of the bait protein in yeast cells, the pGBKT7 bait plasmid was transformed into Y2HGold cells, and subsequently the transformants were grown on SD/-Trp, SD/-Trp/X-α-Gal and SD/-Trp/X-α-Gal/AbA (Fig. 2b). Autoactivation activities from the bait would enable the expression of reporter genes and result in blue colonies on SD/-Trp/X-α-Gal and SD/-Trp/X-α-Gal/AbA plates. The results showed that no autoactivation activity was detected from TgGRA15, as shown in Fig. 2b. Furthermore, the colony size of Y2HGold transformed with bait plasmid was similar to that of Y2HGold transformed with the empty pGBKT7 vector. Based on these results, the pGBKT7-TgGRA15 bait plasmid was used in the Y2H screening.

### Y2H screening against a mouse cDNA library using TgGRA15 as bait

**Fig. 3:**
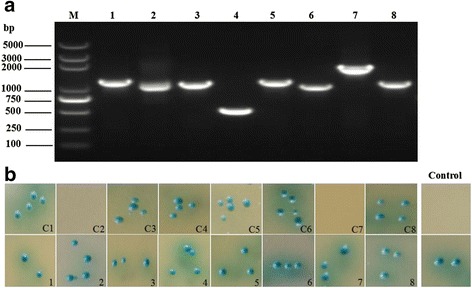
Analysis of putatively positive clones. **a** Gel electrophoresis of PCR products amplified from putatively positive prey plasmids. Lane M: DL5000 DNA Marker; Lanes 1–8: eight blue clones containing prey1–8 (prey plasmid isolated from eight blue clones), respectively, were amplified by T7 sequencing primer and 3'AD sequencing primer. **b** Confirmation of putatively positive clones; C1–C8: zygotes formed from Y187 cells containing Prey1–8 mating with Y2HGold containing empty pGBKT7 vector, respectively. 1–8: zygotes formed from Y187 cells containing Prey1-8 mating with Y2HGold containing pGBKT7-GRA15 bait plasmid, respectively. Control, Y2HGold transformed with pGBKT7-53 or empty pGBKT7 vector were used to mate with Y187 cells containing pGADT7-T, respectively. The mated cultures were then grown on QDO/X/A agar plates, respectively

To screen host proteins that interplay with the GRA15 bait by Y2H system, Y2HGold cells containing the pGBKT7-TgGRA15 plasmid were used to mate with Y187 cells harboring the Mate & Plate™ Universal Mouse (Normalized) cDNA library. Based on the number of colonies on different selection plates, the mating efficiency was calculated to be 6.8% and 5 × 10^6^ colonies were screened. Among 72 colonies (2–3 mm) grown on DDO/X/A plates, 18 were blue. Subsequently, these 18 blue clones were patched out onto higher stringency QDO/X/A agar plates. As a result, eight out of the 18 colonies still turned blue. The prey plasmids were then isolated from these potential positive interactors and rescued by transformation into *E. coli* DH5α competent cells. The specific insert on each prey plasmid was PCR amplified using T7 sequencing primer and 3'AD sequencing primer. The results of PCR amplification are shown in Fig. 3a. To confirm the specificity of interaction, Y187 strain transformed with each potential positive prey plasmid (preys 1–8) was used to mate with Y2HGold containing pGBKT7-TgGRA15 bait plasmid. The results showed that all of the zygotes resulted from mating turned blue on QDO/X/A plates (Fig. 3b, 1–8). To eliminate the false positive hits, Y187 strain transformed with each potential positive prey plasmid (preys 1–8) was then used to mate with Y2HGold containing empty pGBKT7 vector. In this case, C2 and C7 showed no colony on QDO/X/A plates, whereas the others still turned blue (Fig. 3b). The control of Y187 containing pGADT7-T mated with Y2HGold containing pGBKT7-53 showed blue colonies, whereas mating with Y2HGold containing empty pGBKT7 vector showed no colony on QDO/X/A plates. Compared to the results of the control, two host proteins were identified to interact with TgGRA15.

### Sequencing and analysis of positive prey

To determine the identity of positive clones, the two prey plasmids were sequenced using T7 primer. The sequencing results were analyzed by Basic Local Alignment Search Tool (BLAST) in NCBI, which revealed that the two inserts had 100% sequence identity to that of the two *Mus musculus* genes: the expressed sequence AW209491 (GenBank accession nos. AW209491 and NM_134067.4) and the leucine zipper protein 1 (Luzp1, GenBank accession no. BC053451.1). The sequencing results also showed that the AW209491 containing hit encoded a 100-residue fragment fused to the GAL4 DNA-binding domain, and the Luzp1 containing hit encoded a 104-residue region. These two proteins were not previously reported to interact with TgGRA15. Luzp1 is an annotated protein while AW209491 is of unknown function. The biological process analysis of Luzp1 revealed that Luzp1 is related to system development (artery development, neural fold bending and ventricular septum development) in the biological process. Also, in the cellular component ontology, Luzp1 was related to extracellular region and nucleus.

## Discussion

TgGRA15 is involved in several critical functions, including activation of the NF-κB signaling pathway. Despite its importance, information about the host binding partners of TgGRA15 is lacking. To our knowledge, this report reveals for the first time two host proteins that interact with TgGRA15. The study was focused on TgGRA15 of Type II (not Type I and Type III) because Type II is more efficient in the activation of NF-κB, an important host signaling pathway that regulates inflammatory, immune and antiapoptotic responses [11].

Structure analysis of TgGRA15 showed that it contains a transmembrane domain and a PEXEL-like motif (Fig. 1a), a sorting signal that directs *Plasmodium* proteins into erythrocytes, whereas the *T. gondii* PEXEL-like motif contributes to PVM association [31]. In the present study, the 1,056 bp fragment encoding a 352-residue (from aa199 to aa550) peptide of TgGRA15 without the transmembrane domain and the PEXEL-like motif was cloned into pGBKT7 vector as the bait plasmid, followed by screening the host cell molecules that interact with TgGRA15 Type II by Y2H assay. Our results revealed two mouse proteins, AW209491 and Luzp1 that interact with TgGRA15.

Previous studies demonstrated that Luzp, a leucine zipper motif-containing protein, harbors three nuclear localizing signals and numerous putative Ser/Thr phosphorylation sites [32]. Luzp is mainly expressed in the brain, and the knockout of Luzp can lead to failure of cranial neural tube closure, indicating that Luzp plays an important role in the development of embryonic brain [32, 33]. Although there are three proteins, Luzp1, Luzp2 and Luzp4 in mice, the “Luzp” aforementioned is mapped to mouse chromosome 4 [32], which is consistent with the distribution of Luzp1, but not Luzp2 and Luzp4, which were mapped to Chromosome 7 and Chromosome X, respectively. Luzp1 can serve as a bridge factor between the Ada-Two-A-containing histone acetyltransferase (ATAC) and the Mediator complex (MED), and as a result a highly stable meta-coactivator complex (MECO) formed, which is a regulator of a subset of non-coding RNA genes [34].

GRA15 was reported to be detected at the PV, as well as host cell cytoplasm and host cell nucleus [11, 35], however, there is no evidence for how GRA15 targets the host cell nucleus. In addition, structure analysis of GRA15 showed no nuclear localization signal [35]. Given that Luzp1 interacts with TgGRA15, we speculate that TgGRA15 may target the host cell nucleus by interacting with Luzp1. This finding provides a basis for how TgGRA15 translocates to host cell nucleus.

As yeast is a eukaryote cell, proteins encoded by yeast expression system are likely to be similar to their natively expressed ones. Therefore, Y2H system can be an effective and accurate method for large-scale screening of candidate proteins interacting with the bait, and in our study, we have identified two host proteins interacting with TgGRA15. There are probably other host proteins that also interact with TgGRA15, but were not detected in our screening, probably because the Universal Mouse (Normalized) cDNA library may not contain the clones that express potential interactors in the correct manner, due to a reading-frame shift. In addition, as the bait used is a 352-residue, the potential interactors may interact with other regions of TgGRA15. It is also possible, that some host proteins were not identified either due to the host protein not being present in the library or due to the inability of the protein to interact due to the limitations of the Y2H system. Further work is needed in order to evaluate the importance of the interactions described here with regards to their possible roles in *T. gondii* replication or virulence, as our study was limited to identification of proteins in the Y2H, and there is the possibility that some of these protein interactions are not occurring in cells that are infected with the parasite. Nevertheless, the identification of two cellular factors potentially interacting with TgGRA15 during *T. gondii* infection is critical to elucidate processes known to involve TgGRA15 as well as to provide some new clues for studying the biological function of TgGRA15.

## Conclusions

In conclusion, we identified two mouse proteins, Luzp1 and AW209491, that interact with TgGRA15. Luzp1 is a nucleus-targeted protein which is related to system development in the biological process ontology and involved in the regulation of a subset of host non-coding RNA genes. Due to the limitations of the Y2H system, the proteins identified are not exhaustive and interactions identified need to be confirmed by independent experimental approaches in the context of *T. gondii*-infected cells before making any definitive conclusion on the relevance for the parasite life-cycle. Understanding the specific mechanisms underlying TgGRA15 interaction with host partner proteins and the effect of this interaction on the regulation of the NF-kB signaling may lead to the identification of novel therapeutic strategies for the prevention and treatment of *T. gondii* infection.

## Abbreviations

AIDS: Acquired immunodeficiency syndrome
BLAST: Basic Local Alignment Search Tool
cDNA: complementary deoxyribonucleic acid
GBP1: guanylate binding protein 1
GO: Gene Ontology
IKK: IκB kinase
Luzp1: Leucine zipper protein 1
NCBI: National Center for Biotechnology Information
NF-κB: Nuclear factor kappa B
PCR: Polymerase chain reaction
PRU: Prugniaud
PV: Parasitophorous vacuole
ROPs: Rhoptry proteins
TgGRA15: *T. gondii* dense granule protein 15
Y2H: Yeast 2-hybrid
